# Political Disengagement Among Youth: A Comparison Between 2011 and 2020

**DOI:** 10.3389/fpsyg.2022.809432

**Published:** 2022-04-26

**Authors:** Weiyu Zhang

**Affiliations:** Department of Communications and New Media, National University of Singapore, Singapore, Singapore

**Keywords:** disengagement, disinterest, generation, new media, Singapore, youth

## Abstract

This study answers one general question using a country case: *what shapes the young generations’ political disengagement in Singapore*? Taking the generational differences and institutional influence perspectives, this study highlights the time dimension to show the ebb and flow of political and (new) media landscape changes in a non-Western context, Singapore. By comparing focus group discussions conducted among 19–30 years old in 2011 vs. 2020, this paper finds that despite similarly claiming disinterest in politics, the 2011 youth were more attentive to political news than the 2020 youth. The changes in political institutions gave rise to this increased situational engagement. However, the gap between paying attention and taking action was still large in 2020, or even larger than in 2011, due to the increased complexity and competitiveness of politics that the 2011 youth observed *via* social media. The persistence of political disinterest suggests its dispositional connections to psychological barriers that are socially constructed over generations.

## Introduction

Political participation is “at the heart of democracy” (Verba et al., 1995, p. 1) and political interest is the “civic foundation” for participation (Prior, 2018, p. 360). It is claimed that only democracy can “offer citizens opportunities to participate in their own governance” (Rosenstone and Hansen, 1993, p. 1), providing the mechanisms by which citizens can seek to satisfy their needs and preferences. If political interest and participation are so critical to both governments and civil societies, why do citizens choose not to participate and claim no interest in political activities? If non-participation is the simple opposite of participation and lack of interest the opposite of presence of interest, we may expect that political disengagement can be explained by the absence of the factors that contribute to engagement, including motivations, capabilities, and opportunities (Verba et al., 1995). However, the long-lasting phenomenon that a substantial portion of any populations shows little interest to join “the self-governing class” points to the dispositional roots that cause political disengagement (Prior, 2018, p. 353). A simple question remains unanswered: are there any deeper causes that are present in citizens’ life situations that actively encourage disengagement?

When it comes to political disengagement, youth is always a concern for two reasons: first, youth is found to be generally less interested than the elders in engaging in almost every established form of political activities in various countries. There is a world-wide observation that younger generations are less interested in traditional politics and perform fewer classic political activities (e.g., vote). The pattern is consistently found in both developed and developing countries, as well as established and emerging democracies. Meanwhile however, youth leads a new wave of collective actions that are not found or encouraged in the traditional institutions. Youth have been embracing many new forms of politics such as individualized activism (Bosch, 2017), political consumerism (Stolle et al., 2010) and new media based participation (Zhang, 2005, 2013; Vromen et al., 2015). The diverging observations invite scholars to ponder on the dichotomy view on activism vs. passivity (Amnå and Ekman, 2014) and suggest that youth’s participatory practices “take the form of informal, individualized and everyday activities” (Harris et al., 2010). These changes are best observed over time instead of looking at momentary snapshots. However, theorization and empirical examination of political disengagement that takes a longitudinal approach still remain scarce.

This study tries to answer a general question using a country case: *what shapes the young generations’ political disengagement in Singapore?* Taking the generational differences and institutional influence perspectives, this study highlights the time dimension to show the ebb and flow of political and (new) media landscape changes in a non-Western context, Singapore. By comparing focus group discussions conducted among 19–30 years old in 2011 vs. 2020, this paper finds that despite similarly claiming disinterest in politics, the 2011 youth show some differences compared to the 2020 youth. The changes in political institutions such as party competitiveness and social media served as the historical environment that gave rise to these differences. The persistence of political disinterest suggests its dispositional connections to psychological barriers that are socially constructed over generations. This paper concludes with a discussion about broadening the conception of politics and a call for using new media as living spaces for youth to experience politics.

## Literature Review

Empirical evidences once and again show that younger generations are indeed less engaged in established political activities than older generations. Youth’s lower participation in formal politics such as elections has worried many politicians and scholars. Amnå and Ekman (2014), however, argue that political disengagement is not always a threat to democracy. Citizens who look passive can differ significantly in their civic dispositions: the standby citizens are ready to be activated; the disengaged citizens are not making actions but paying close attention to civic issues; and the disillusioned citizens are genuinely passive. As Prior (2018, p. 4) put it, political interest as a disposition takes a long time to develop and most experiences of situational interest never develop into dispositional interest. These different categories of disengaged citizens are found to be different in their strength of the dispositional interest in politics. The genuinely passive citizens have higher psychological barriers as they lose faith in the political institutions. In contrast, the standby citizens have much lower psychological barriers because they are waiting for an opportunity to be activated. This thread of research suggests that we need to go deep in understanding the various psychological reasons behind youth disengagement in politics.

While formal politics seems to lose its appeal to the younger people, alter-activism (Juris and Pleyers, 2009), issue politics (Stolle et al., 2010), identity politics (Marsh et al., 2006) and lifestyle politics (Bennett, 1998) are on the rise, broadening the definition of politics. These alternative formats of politics suggest that the existing conception of politics is too narrow or limited to capture the lived experience of youth (Harris et al., 2010). The narrow vs. broad definitions of politics differ in significant ways. Firstly, political participation is traditionally understood as an obligation for citizens, emphasizing the dutiful role citizens have to serve. Lifestyle politics, in contrast, tends to see personal choices as the foundation of political actions. Options of lifestyles and consumptions are taken by citizens to express their political views, not to fulfill duties. Secondly, formal politics often assumes a clear differentiation between the public and the private, and discourages personal issues from entering the political agenda. However, identity politics that foregrounds personal characteristics such as gender and race dissolves the boundary and allows youth to explore their identity construction. Thirdly, youth may not be interested in the grand politics but can be drawn into politics based on specific interests. Those who care about certain issues such as environmentalism are not necessarily into party politics, for instance. Lastly, traditional politics is imagined within the national borders. Globalization, facilitated by new information and communication technologies, has led to worldwide networks of participation among young alter-activists. This thread of research suggests that youth disengagement in traditional or formal politics indicates the rising of a new era of politics, defined and practiced by the young generation themselves.

Both lines of thoughts point to two steps of theoretical explanations of political disengagement among youth: the first step is to understand the distinctive features, especially their psychological dispositions, of the young generations (i.e., generational differences), and the second step is to discover the surrounding environment that shapes the distinctive psychologies (i.e., institutional influence). The following section presents overviews of the key arguments and debates that follow these two steps of explanations.

### From Generational Differences to Institutional Influence

Among early efforts that try to explain the lack of political engagement among younger generations, life cycle theory suggests that younger people do not involve in politics as much as older people because they are in a unique life stage which exposes them to all kinds of starting-up problems (Zukin et al., 2006, p. 11). There is a transition problem if younger people do not take up the mainstream forms of political participation as they grow older. Generational theory counter-claims that every generation grows up in distinctive environments, in which unique events and trends shape the generation’s political subjectivities (Wyn and Woodman, 2006). As results, we see very different patterns in different generations regarding their interests and participation in politics (Adsett, 2003). One view under the generational theory sees the current generation of youth as “radical unpoliticals” (Farthing, 2010) who reject the regulatory model of politics (Manning, 2010) deliberatively and embrace the new politics of fun. As much as we can agree that generations are different, what often follows this consensus is the disagreement on what shapes these distinctive characters. The examination of institutional influence comes from the tradition of comparative politics and puts its focus on structural factors that both constrain and enable the generational distinctions.

The institutional influence approach to understanding the formation of any political characters comes from the long tradition of political socialization studies. Political socialization emphasizes the factors that influence the development of one’s political attitudes, knowledge, and identity in one’s formative years. Works on political socialization often focus on the role of family communication patterns, specifically, how parents’ political predispositions are transmitted to their children (Bacovsky and Fitzgerald, 2021; Siegel-Stechler, 2021). Political socialization is also expanded to other interpersonal communication such as that between children/adolescents and their friends, peer groups, etc. (Gordon and Taft, 2011). Along the same line, civic education works examine how schools and formal education influence children/adolescents’ political socialization (Kupchik and Catlaw, 2015). Media scholars add that not only purposeful and active learning can shape political cognitions and behaviors, but also passive consumption of information from media is able to affect political socialization. One relevant piece of finding is that the significant role of news consumption in fostering political participation has been widely supported (e.g., Quintelier, 2015).

The institutional influence view echoes with the concepts such as opportunity structure in drawing our attention to the environment that surrounds youth when they grow up. Frist of all, it is the political system that opens up certain opportunities while closes others for citizens to take part in political decision-making. As Marsh et al. (2006, p. 5) argued, political apathy is “rather a problem pf political exclusion, with many alienated from a political system which they experience as unequal and unfair.” However, one limitation of the institutional view is its lack of recognition of changes, or the tendency to form a fixed understanding about one particular country and its institution. It is hard to observe how institutional changes within one country shape youth’s political disengagement if our data points are snapshots of particular moments. With the longitudinal research design, this paper is able to answer the following research question (RQ1)*: how do the institutional changes influence the generational differences of youth in Singapore?* The following section discusses new media as an institution of living space.

### The Changing Landscape of (New) Media

Terms such as digital natives suggest that the younger generations were born or grew up in a world that is wired by the Internet, implying that new media are one of the defining distinctions that previous generations do not have. However, the same presence of new media in youth’s lives does not translate to the same influence of new media on youth participation. For instance, the Danish Millennials in Andersen et al. (2020, p. 133) study was described as “the Lost-in-Transition Generation” who “get most of their political information from social media but are not politically mobilized at all.” In contrast, the US Millennials in Milkman’s review (Milkman, 2017, p. 5) “comprise the bulk of those involved in the new movements that emerged on the Left” in the post-2008 period such as the 2011 Occupy Wall Street and their activism is featured by their “unprecedented use of social media.” It is clear that generations with same labels are not the same across countries, making it necessary to understand why the influence of new media differs for the same generations in different contexts.

The institutional influence view prompts us to think of new media as a political socialization institution. How new media function as an institution could be examined through two ways: one is to treat new media as an institution that produces and disseminates information, and ask how this information source influences political knowledge, interest, efficacy and political participation. The second way to study the institution of new media is to consider new media as venues for political participation, such as online petition sites, online activist groups, political discussions in forums and on social media, and Internet tools used for mobilizing and organizing offline actions. However, it is important to bear in mind that not all information circulated on new media is meant to motivate political engagement and not all online spaces are for civic purposes. Some recent changes in the past decade have exacerbated the problems with new media.

One change that has gradually happened was the normalization of the Internet, with commercial and political forces wielding disproportionally control over our digital life. The recent wave of algorithm-driven platforms that fed into human weaknesses (e.g., confirmation biases, intolerance and hatred) is an adequate illustration to show how commercial interest can be contradicting important civic values. The information found in new media has deviated far from the utopian ideal of the marketplace of ideas and become shaped or distorted by well-resourced actors. Disinformation driven by partisan fanatics, foreign propaganda, or the states themselves is a world phenomenon. The civic spaces online have been squeezed: checking friends’ social media updates, watching long and short videos for fun, and playing all kinds of digital games have taken the majority of users’ free time. We therefore need to examine new media as part of youth’s everyday life, emphasizing both the opportunities and threats brought by residing in a digital world. For instance, Middaugh et al. (2017) found that interest-driven online communities are different from friendship-driven communities, as youths who participated in the former experienced more conflicts than those in the latter.

When we see new media as an institution of living space, its influence on political participation becomes more encompassing than information sources or action tools. New media provide diverse spaces to live in, while political spaces are only a small number of options. These diverse spaces have fluid boundary, while the personal spaces can become political or vice versa. Personal interests in certain issues may turn into political actions at a global scale, with the help of the Internet-enabled networks. The second research question (RQ2) this paper tries to answer is: *how do new media exert their institutional influence on youth disengagement over time in Singapore?* The following section introduces the research context, highlighting the specific changes in the political system and media landscape.

### Research Context

As Singapore obtained independence only in 1965, the Singapore government has used Pioneer and Merdeka generations to refer to Singaporeans who had some life experience living in a British colony and contributed to the early years of nation-building. When the Pioneer and Merdeka generations had first-hand experience with poverty or wars, people who were born since the late 1970s grew up in a fast changing Singapore that quickly turned from a third world country to a first world one. When the late-1970s still constantly hear from their parents and grandparents how they have made real an economic miracle, people who were born around the late 1980s grew up in a *de facto* high-performance economy, with computers and the Internet ubiquitous in their daily life. Internet access increased from 78% in 2010 (Infocomm Development Authority, 2010) to 98% in 2019.

The institutional changes regarding political participation were parallel to the economic growth, from a development stage to a post-development stage to a globalization stage. As a former British colony, Singapore’s election system started from a simple plurality in single-member constituencies and changed through a set of reforms in the 1980s (Li and Elklit, 1999; Tan, 2013). Despite that the ruling party kept wining general elections with majority votes (60% and more), the competition from the opposition parties continued to grow stronger. Although voting is compulsory in Singapore, there were still uncontested constituencies in the 2011 General Election (GE, the most important election that generates parliament members), which means that these constituencies only had the People’s Action Party (PAP, the ruling party) candidates. Residents living in those walkover constituencies, therefore, did not get a chance to cast a vote. The election scene has changed drastically since 2011. In both GE2015 and GE2020, all constituencies were contested. The numbers of parties and candidates who joined GE2020 were historically highest. The political transition in Singapore over the last decade suggests a counter phenomenon to Mayhew (1974) varnishing marginal—the likelihood to vote for the incumbent was generally declining, although with an unusual spike in GE2015.

Social institutions such as schools have gone through major changes, too. In the early years of the young nation, schools emphasized “education for living,” a clear indicator of the survival mentality (Chia, 2016). “Asian values” that stress national cohesion among multiple cultures dominated the educational discourse till the end of the 20th century. In 2001, Social Studies as a compulsory and examinable subject was introduced in Singapore secondary schools as the curriculum of citizenship education (Sim and Print, 2005). Co-curricular activities (CCA) are compulsory, non-academic activities that aim to encourage students’ holistic development in life skills, competencies and values. Both subjects provide the youth opportunities to engage in political understanding, community volunteering and self-organization. Starting from the 21st century, schools prioritized education programs that tackle the challenges brought by globalization such as bringing in migrants while not diluting the national identity.

Singapore’s communication ecosystem is characterized by the differentiation between mainstream media vs. alternative media. Historically, mainstream media in Singapore were established along with the nation, supported by the government in various ways (e.g., there was a radio and TV license fee for residential owners until 2011). Mainstream media enjoy an almost monopoly in the local newspaper, TV, and radio broadcasting industry, mostly aligning with the governmental agenda. Alternative media before the Internet took the format of brochures, printed handouts, and books (some banned by the government). The Internet and mobile phones gave rise to the online sphere for voices critical of the government, in venues such as opposition party websites, citizen journalism projects, blogs, Facebook pages, Twitter accounts, YouTube channels, and even email lists. The use of traditional mass media such as print newspapers has been steadily declining but their online versions are still popular among the citizens (Soon, 2020). Online only media, including social network sites, have become the top media used by younger generations (Zhang, 2016). The last decade witnessed this gradual and uneven procedure of online media taking over offline media in becoming the most important information source for Singaporean youth.

## Materials and Methods

This study employs focus group discussions with Singaporean youth, defined as 15–35 years old by Singapore’s National Youth Council (2021). To conform to our institutional review board’s requirements on age limit (18 years and older), our participants’ age ranged from 19 to 30 in 2011 and from 18 to 30 in 2020. The groups had an average of 4–7 participants and lasted from 1.5 to 2.5 h. In each year, we recruited 62 participants (12 groups; on average five participants per group) through online advertisements and snowballing from the earlier participants. They received two movie vouchers (in 2011) or Singapore dollars 40 (in 2020) for their participation. Table 1 summarizes basic comparisons of the samples between the 2 years. The recruited participants all had low level of political interest: their average interest in politics was about 2 (*SD* = 0.74 in 2011; 0.48 in 2020) in a four-point scale, corresponding to “not very interested.” They were avid and long-term Internet users: in both cohorts, an average of 12 years of Internet use was reported and almost every participant said they used the Internet several times a day. The group discussions were all conducted in English. In 2011, face-to-face discussions were conducted at a classroom located in a large university in Singapore. In 2020, the pandemic made such physical groups impossible and we relied on Zoom for the discussions. We observed no significant differences between the online vs. offline mode of group discussions, as in both formats, participants were strangers to each other so followed a rather polite way of interaction. The online mode facilitated participants’ sharing of examples, such as websites.

**Table 1:** Basic statistics about the 2011 vs. 2020 samples.

	2011 Sample	2020 Sample
Age (mean/SD)	23 (3.12)	22 (1.99)
Females (%)	52%	55%
Majority ethnicity (%)	88%	85%
Attention to news about politics and government (mean/SD on a 1–5 Likert scale)	2.05 (0.82)	2.50 (0.83)
– Newspaper days in past week	1.23 (1.56)	1.92 (1.47)
– TV days in past week	0.98 (1.46)	1.72 (1.20)
– Radio days in past week	0.30 (0.68)	1.55 (1.25)
– Internet days in past week	1.46 (1.77)	3.50 (1.91)

Both waves of focus group discussions (FGD) followed a similar procedure: following the online advertisements, the participants were asked to fill out a screening survey in which basic demographics, citizenship status, political interest, attention to news and politics, and preferred time slots were obtained. Eligible criteria include Singaporean citizens, their age being between 15 and 35 and their availability in one of the designated time slots. Eligible participants were invited to join the discussions with logistic details through emails. Before the FGD started, participants signed an informed consent form that contains basic information about the study and their voluntary consent to participation. They were explicitly informed that the discussions were audio-recorded.

In both years, a general election was just held within the last 5 months. Our focus group participants had fresh memory about the elections, even if they were generally not interested in politics. The participants were firstly asked about their understanding and feeling about politics, supported by a set of real-life local examples as prompts. We followed up with a set of questions on their lack of political interest and its underlying reasons (e.g., efficacy, motivation, knowledge, skills, opportunities, political system/culture, and social norms). Our last set of questions focused on new media and their potential impacts on curbing or encouraging political activeness. Several real-life local examples were provided to illustrate problems such as information overloading, filter bubbles, fake news, cyberbullying, envy and depression.

Three (2011) and two (2020) experienced and trained moderators conducted the discussions, who were graduate and undergraduate students from social science majors such as communication and political science. All moderators went through training sessions conducted by the lead author. Training included both topical knowledge about political participation in Singapore and basic methodological concepts such as grounded theory, constant comparative approach, and the three-step coding analysis. The moderators closely followed the discussion guide but was also told to ask follow-up and probing questions when necessary.

The recordings were later transcribed and summarized for analysis. In total, there were about 700 pages of transcribed data. The three-step coding analysis (Tracy, 2019) was conducted: open coding was first applied to a line-by-line analysis, during which unrestricted and recurring ideas and concepts were identified. Second, axial coding was used to group related ideas and concepts to create categories. Third, selective coding combined overlapping categories and refined categories to create themes. Our final themes cover the institutional influence on and psychological barriers to youth engagement, as well as the role played by new media and the future. Additionally, a temporal-comparative analysis was conducted to connect the themes with the changing historical background.

## Results

Before delving into the in-depth analyses, it is worth reporting some basic comparisons between 2011 and 2020. A general trend was that the 2020 sample was more attentive to news about politics and government than the 2011 sample, although both samples reported low interest in politics (average is 2 out of a four-point scale). Moreover, the 2020 sample spent more days in the past week on news about politics and government found in newspapers, radio, TV, and the Internet. The most eye-catching increase was shown in using the Internet for such news, which over the past decade took the leading role from newspapers. However, the higher usage of the Internet does not necessarily mean that its usage can change young users, especially those who have little interest in politics. The following analyses start from comparing the young people’s own definition of and feelings about politics, followed by a discussion on the changing role of social media, and lastly a future-looking section on how these disengaged youth may start becoming active.

### Not My Politics…Yet: Institutional Influence on Generational Differences

In general, most participants from both cohorts associated politics with election, political parties, and political leaders. According to our 2011 participants who lived in the walkover constituencies, since people cannot vote, they cannot be bothered with the election politics. However, as a result of the increased competition in the following general elections, our participants cannot view election as forever irrelevant any more. The 2020 participants either have voted or held the expectation that they will have to make a choice at the ballot booth, when they become 21 years old. This perspective of voting served as a trigger for the youth to start paying temporary attention to politics. As participant 2020-1101D4 put: “Because I think even for my group of friends, we were not too interested in politics until we had to vote. So I guess we would try to look up things about it in order to make an informed choice.” However, they quickly pointed out the lack of participation and interest when election periods are over. Participant 2020-1101E4 commented: “I feel that most Singaporean youths do not really care much about politics and you see people care about politics is mostly seasonal, you know the election period so I think that means that they aren’t actually that interested in politics.”

More often than not, elections and politics were seen as part of a game for power whereby politicians “fight for positions.” Certainly, politicians play an essential role in politics and an association between politics and political figures is inevitable. But in 2011, talking politics almost meant entirely about political figures. The participants back then discussed or even gossiped in length about the powerful elites and their families. One participant from 2011 went further by associating politics with Lee Kuan Yeow (LKY), the founding father of Singapore. Opposition party leaders who openly criticized and challenged the ruling government were seen as “daring,” “sad,” “a little bit pathetic” and “bordering on insanity.” “Generally they are all in trouble.” “They do not win, they always lose.” “Just ignore them. Not, not very high quality one.”

After LKY’s passing that closed a political era in 2015, the 2020 participants rarely mentioned the lass name Lee. Instead, other prominent politicians such as ministers and their performance in elections were frequently described and evaluated. As opposition politicians gained more seats in the parliament in GE2020, the overall tone when talking about opposition party members changed. They used words such as “encouraging,” “relatable,” “politicians that I feel that I can identify with” to describe the younger generation of oppositional party leaders such as Pritam Singh and Jamus Lim. Although there are skeptical views regarding their credentials and popularity, at least these political figures are no longer seen as “crazy.” Another notable change is that our 2020 participants started to include non-party candidates such as issue activists when talking about political figures. Again, they mostly used positive words, such as “cool,” “passionate” and “admirable,” to describe those who advocate for issues such as recycling and climate changes.

However, the improved perception of opposition parties does not help much in convincing our participants to join party politics. On the contrary, the heightened competitiveness among parties and their supporters has become a major turning-off point for youth who dislike conflicts, rivalry, and fights. In participant 2020-1031B1’s words, “inherently it involves conflict at the end of the day because there’re so many different views and people have like their own point of view that they want to like convey. And people like me, I like to avoid conflict.” While our participants claimed disengagement in politics, they almost all had some experience in civic activities such as volunteering. This has to do with the school system that makes CCA compulsory for all secondary school students and gives CCA credit points to college students. But most participants did not see these activities as anything political, a view that did not change over the years. As participant 2020-1031A2 put it, “I feel that as long as I participate in volunteering, no matter what affiliations, as long as the beneficiaries benefit from it, I will find it meaningful. We do help NGOs and other beneficiaries; it is not political in nature.” To summarize our first analyses, Table 2 shows the major findings.

**Table 2:** Summary of analyses of the 2011 and 2020 samples.

	2011 Sample	2020 Sample
Definition of politics	– Narrowly in the sense of party politics and elections.	– Narrowly in the sense of party politics and elections.
Election engagement	– Elections were not competitive. Many youth did not get a chance to vote so paid little attention to electoral information.	– The increased competitiveness in local elections forced youth to pay temporary attention to electoral information but turned off those who are conflict-avoidant.
Community engagement	– Those who were disinterested in politics were (sometime non-voluntarily) engaged in volunteering.	– The denials of engaging in politics signaled the desire to be non-partisan and non-confrontational rather than being apathetic or inactive.
Psychological barriers to engagement	– The positive feeling of satisfaction and contentment of the status quo. – Being fearful of state surveillance and repercussions.	– The positive feeling of satisfaction and contentment of the status quo – Being worried about public scrutiny and criticism. – The increased complexity of politics left youth feeling confused and frustrated.
The role of new media	– Youth turned to “radical” sources for fun. – New media were the only information sources to hear about anti-government voices. – Political debates were done by a vocal minority on the Internet.	– Youth turned to “neutral” and “light” sources for fun. – Social media became the default mainstream to obtain information from both the establishment and their critics. – Social media supported a regularization of political discussions among ordinary Internet users.
Prospective for engagement	– More ready to participate in smaller scale issues that are relevant to their own lives, than making system-level changes.	– More ready to participate in smaller scale issues that are relevant to their own lives, than making system-level changes. – Hope for the system to remove restrictions and create channels that cater to their habits.

### Fear, Frustration, and Satisfaction: Psychological Barriers to Political Engagement

When asking about why our participants are disinterested in politics, they started from describing their feelings about politics in Singapore. Traditionally, they inclined to attribute their negative feelings to the governmental style that tends to be authoritarian. One such negative feeling is fear. In 2011, the participants demonstrated a strong awareness of state surveillance. Some of them believed that votes can be tracked and most of them were certain that online activities can be traced to the individuals. They were worried that their political comments or activities would be linked to other non-political aspects of their life, such as career development. Participant 2011-0289S said: “What if I want to apply for public service next time and they actually go and look at my practical history like, oh actually this guy wanted to rally at Speakers’ Corner, let us not put him in public service.” The feeling of being “worried” and “scared” was still present in 2020 but the source of fear changed from all government-related to more social reasons. Participant 2020-1125D3 talked about this fear of judgement: “In a lecture of 200 people, we might not speak up. Speaking up in a public forum will also not be done. The concept of ‘face’. Keep your head down and work hard.” Moreover, the 2020 participants saw being politicians a “tiring” job that requires them to be “thick-skinned”: they feared of being ridiculed by the public when they make mistakes.

While fear of state surveillance became less prominent in explaining disengagement, other negative feelings emerged in 2020. These negative feelings such as frustration and confusion were triggered by the “messy” and “complicated” nature of politics. The messiness of politics apparently had a connection to the increased competitiveness in local politics. Participant 2020-1031B1 commented: “But there’s like differing views from so many different people, so many different political parties. Then ends up it gets too long to read and like fully understand everything. So, end up, half way I just give up.” Another connotation of messiness refers to the “dirty” or “under table techniques” they thought politicians often use. Feeling “disturbed,” they wanted to “get away from it.”

However, it was not just negative feelings that drove disengagement. In both 2011 and 2020, a consistent explanation attributed their lack of interest in politics to the social and economic stability in Singapore, implying a sense of satisfaction. In 2011, participants referred to the economic miracle as an achievement of the ruling party, which is the only government that has a proven record of managing the country well. “We will not be stupid to rock the boat,” participant 2011-0012T said. In 2020, Singapore’s economic growth was no longer the focus of relative advantage but our participants still showed contentment regarding other aspects of the governance. Participant 2020-1031F1 referred to the recent pandemic: “I’m actually very fortunate to actually like (be) able to live here and like all the measures that they come up with, promptly, to actually fight this COVID-19…As of now, I would not really question like, why they made this decision.” See Table 2 for the summary of the analyses.

### Light and Fun: New Media as Living Spaces

Regarding the mainstream media, the consensus among our participants over the years was that they adopt a pro-government agenda. The lack of diversity in mainstream media coverage of political issues, to some participants, makes it “boring” to follow, let alone participating in. Therefore, new media have caught the attention of many youth. In 2011, the online alternative media were blogs written by individual commentators and citizen journalism websites maintained by a group of semi-professional writers. Our participants visited these online venues for the other side of the story, but more often than not, for fun.

This entertainment-seeking motive persisted into 2020, despite that the online venues have changed a lot. The new online-only local news sources have presented political information in a “light” way that attracted the youth. Memes, in the formats of funny stickers, short video clips, and simple infographics, have replaced blogs and websites to be the major sources of fun in 2020. Compared to 2011, local politicians have levelled up their social media presence: almost every politician set up Facebook pages, twitter accounts, or Instagram channels. The participants, however, pointed out that politicians with the intention to reach youth through social media risks trivializing politics. For instance, a local politician showing a video of himself eating flowers or speaking the jargon of youth attracted lots of views, likes and shares. Some participants thought that supporting a candidate simply because of his/her online popularity is a manifestation of political apathy, too.

The Internet has evolved from a space for information exposure into a world of social networks. Even social network sites (SNSs) themselves have changed significantly within the last decade. Facebook already reached 77% of Singapore’s online population in 2011 (Infocomm Media Development Authority, 2020). Back then, there were already politicians holding Facebook accounts, and individuals voicing their opinions on their news feeds. Our 2011 participants, however, were cautious about sharing their political views on Facebook, citing cases that have been sued and punished due to their online speeches. In 2020, sharing oppositional views on SNSs has become common, although new laws are in place to control online speech. Participant 2020-1031G1 described: “For me, on Facebook, actually I see more of the opposition view than the PAP view.” Another obvious change was the rise of visual-centered SNSs such as Instagram. What one of our 2011 participants wished for, 10-min videos to introduce political candidates, has been the reality in 2020. These visual materials are even shorter and simpler than Facebook posts and blog articles, which make the 2020 participants think that they can always come back to know more when they are a bit freer. See Table 2 for the summary of the analyses.

### I Will Participate If: A New Conception of Politics

Our results unveil some consistent finings regarding what may trigger youth to participate in politics. In both 2011 and 2020, making real changes will be the strongest motivation. However, the political system seemed to be too stubborn to change. Although GE2020 was seen as a major improvement for the opposition, participant 2020-1125D1 wasn’t optimistic about changes and said: “So even if we let opposition into the parliament but the majority is still be like PAP. So in the end, there’s like nothing that we can change to the political system in Singapore.” Hoping to see tangible changes they can actually make an influence on, some participants turned to volunteering and specific policies. For instance, the 2020 participants cited recent cases in which social media were used as a major channel to rally support and push for changes in school policies. Such micro-changes have captured our 2020 participants’ interest, and resulted in occasional participation in such activities.

The question then becomes, which kind of micro-changes our participants will envision themselves taking action upon? The answer is mostly the relevance of an issue. Back to the petition to adjust bus fare for polytechnic students in 2008, many 2011 participants shared that they signed the online petition because it directly affected them. Some participants explained that when they started working and paying taxes, they would be more active in having a say in their communities. One important way to determine relevance is whether the issue is prominent in their social circles, with the participants taking cues from social media. Direct invitations from friends and family members often lead to their occasional participation. Role models certainly helped, especially those who came from similar age groups and background.

When asking about particular ways to get youth participate more in politics, our participants suggested two approaches: remove barriers and create channels. The former answer was more seen in 2011 and the latter more in 2020. One persistent barrier to join politics is again, politics’ inherent nature of being complicated. A second barrier refers to the systematic restrictions on participation in the country. Our participants hoped the system can be more open and transparent than how it is now. By creating channels, several participants in 2020 asked for direct interaction in a dialogue format with key politicians such as ministers. One of them pointed out that there should be “middlemen” who can provide such a platform for dialogues. Another participant suggested that universities can organize such sessions as part of civic education. See Table 2 for the summary of the analyses.

## Conclusion and Discussion

Our longitudinal research design allows the study to show that the changes in our youth participants from 2011 to 2020 were slow but real. A time-series component in our qualitative study reveals subtle differences deeply rooted in psychology, which could be easily buried in quantitate standardized data such as a survey question on political interest. Although lack of motivation is persistently strong because of a widespread satisfaction with the status quo, other psychological factors that actively discourage youth engagement changed in their weights of impact. The fear of government surveillance has lessened and the fear of public criticism has increased among youth. To answer RQ1, the political institutional changes led to observable differences between the 2011 and 2020 youth, but the differences lied in psychological motivations and perceptions more than actual participation acts. The full immersion in the social media space also meant youth took in all kinds of information from the Internet in 2020, not limited to the pro-opposition messages from a minority of online vocal opinion leaders in 2011. Social media that allow conflicting voices from almost everybody, however, overwhelmed the 2020 participants. To answer RQ2, new media changed from an institution of information sources to an institution of living spaces, where light and fun activities within one’s social circles were preferred over conflictual political engagement with disagreeing strangers.

The opening of some institutional opportunities did not fully translate to interest and participation, due to the presence of factors that Verba’s model (Verba et al., 1995) has not paid sufficient attention to. The finding illustrates that situational interest, made possible by opportunity windows such as competitive elections, is hard to translate into dispositional interest. Psychological barriers, such as the strong preference for conflict avoidance, cannot be explained away by only political reasons. There is a deeper association with psychological disposition and social norms that pre-conditioned our participants’ comfortable way of engagement, which tends to be non-confrontational and of small scale. In order to address these psychological barriers, both the social and the political institutions need to put in efforts. When it comes to shaping social norms, media as a political socialization institution needs to be understood as not only information sources but also spaces in which youth encounter other members of the public and observe how (or not) to be an active citizen. Social media, for example, not only confused the youth with conflicting information but also frustrated them when other fellow citizens demonstrated intolerance and incivility towards disagreeing members.

In addition, the paper contributes to the generational perspective of civic (dis)engagement, by showing that the same generation defined in age groups is far from homogeneous. The heterogeneity within one generation is illustrated in at least two ways: first, the engaged and the disengaged members from the same generation interacted with the changing environment in different ways; second, the same generation from different countries have experienced changing environments that both have similarities (e.g., the rise of social media) and sufficient differences (e.g., political systems). While generations are still helpful concepts that make explicit the events and trends that are historically embedded, we need to unpack the actual connotations of generational labels when examining certain groups and countries.

All studies have their limitations and this one is no exception. First, the study aims to find out reasons behind why youth is disengaged in formal politics and thus, adopts a rather limited conception of politics. Our participants explicitly said that they were not that interested in politics, which turns out to be mainly party politics. As the findings suggest that the participants were not as disengaged as they claimed so, future studies can adopt the broad conception of politics and investigate its practice among youth. Second, we were interested in the political socialization process that includes years preceding the legal voting age of 21 but we partially relied on participants’ memory to understand those years. Future studies should include adolescents younger than 18, who are experiencing these primitive years, to understand the ongoing process. Third, our two groups of participants were similar but not identical. Tracking the same group of youth as they grow old might help to better understand changes. Lastly, as a single country study, the generalization of our findings needs to consider the country context. We suggest that the cluster of countries that has gone through both economic and political transition rather peacefully after World War II may resonate with our findings.

The paper concludes with a discussion about broadening the conception of politics and a call for using new media as living spaces for youth to experience politics. In Singapore, the 2020 participants who claimed disinterest in party politics actually demonstrated periodical attention to elections and some knowledge of political issues through mostly social media usage. This suggests that a new era of politics is emerging from youth’s lived experience. Personalized or individualized forms of participation at small scales put more emphasis on identity politics and issue politics as lifestyle options rather than party politics as civic obligations. Youth’s politics also happens more in alternative spaces such as new media rather than parliaments. Taking this broadened conception of politics helps us to understand how future politics will look like. For other societies in political transition such as the post-Soviet nations (e.g., Allaste and Cairns, 2016), the Singapore case shows that despite the historical legacies, the experiences of situational engagement in both formal and alternative politics will accumulate over time as the political situations that require youth’s participation occur more often. Social media can open the windows for alternative politics in transitional societies as demonstrated in both online campaigns (Bosch, 2017) and daily participation (Zhang, 2005, 2013). If social norms that pre-condition psychological barriers can be changed (e.g., societies can be more tolerant of disagreements and conflicts), it is a matter of tipping point for some of the disengaged youth, especially those who fall under the standby citizens, to start engaging in larger-scale public causes. A new generation of political leaders, such as role models from their own peers, may inspire these youth into actions that aim for systematic changes. For disinterested youth in non-transitional societies, many countries have employed institutional changes to drive participation (e.g., lowering voting age). Our study suggests that on top of political institutions, psychological factors that are rooted in social norms should be understood and addressed. Creating both online (e.g., dedicated platforms for citizen debates) and offline spaces (e.g., dialogues with key politicians) that can tackle the psychological barriers and reform the social norms is the way to build interest through everyday experience in the lifeworld.

## Data Availability Statement

The raw data supporting the conclusions of this article will be made available by the author, without undue reservation.

## Ethics Statement

The studies involving human participants were reviewed and approved by DERC, National University of Singapore. The patients/participants provided their written informed consent to participate in this study.

## Author Contributions

The author confirms being the sole contributor of this work and has approved it for publication.

## Author Disclaimer

Views expressed are those of the author alone and do not necessarily reflect opinions of the sponsoring agencies.

## Funding

This work was supported by National University of Singapore under HSS Collaborative Seed Grant R-124-000-112-646, NUS-University of Sydney Collaborative Grant R-124-000-102-133, and the International Development Research Center, Canada under Grant R-124-000-037-597.

## Conflict of Interest

The author declares that the research was conducted in the absence of any commercial or financial relationships that could be construed as a potential conflict of interest.

## Publisher’s Note

All claims expressed in this article are solely those of the authors and do not necessarily represent those of their affiliated organizations, or those of the publisher, the editors and the reviewers. Any product that may be evaluated in this article, or claim that may be made by its manufacturer, is not guaranteed or endorsed by the publisher.
